# Population genetic structure of *Anopheles gambiae *mosquitoes on Lake Victoria islands, west Kenya

**DOI:** 10.1186/1475-2875-3-48

**Published:** 2004-12-06

**Authors:** Hong Chen, Noboru Minakawa, John Beier, Guiyun Yan

**Affiliations:** 1 Department of Biological Sciences, State University of New York, Buffalo, NY 14260, USA; 2 Global Public Health Research Group, Department of Epidemiology and Public Health, University of Miami School of Medicine, Miami, FL 33136, USA

## Abstract

**Background:**

Understanding the genetic structure of island *Anopheles gambiae *populations is important for the current tactics in mosquito control and for the proposed strategy using genetically-modified mosquitoes (GMM). Genetically-isolated mosquito populations on islands are a potential site for testing GMM. The objective of this study was to determine the genetic structure of *A. gambiae *populations on the islands in Lake Victoria, western Kenya.

**Methods:**

The genetic diversity and the population genetic structures of 13 *A. gambiae *populations from five islands on Lake Victoria and six villages from the surrounding mainland area in the Suba District were examined using six microsatellite markers. The distance range of sampling sites varied between 2.5 and 35.1 km.

**Results:**

A similar level of genetic diversity between island mosquito populations and adjacent mainland populations was found. The average number of alleles per locus was 7.3 for the island populations and 6.8 for the mainland populations. The average observed heterozygosity was 0.32 and 0.28 for the island and mainland populations, respectively. A low but statistically significant genetic structure was detected among the island populations (F_ST _= 0.019) and between the island and mainland populations (F_ST _= 0.003). A total of 12 private alleles were found, and nine of them were from the island populations.

**Conclusion:**

A level of genetic differentiation between the island and mainland populations was found. Large extent of gene flow between the island and mainland mosquito populations may result from wind- or human-assisted dispersal. Should the islands on Lake Victoria be used as a trial site for the release program of GMM, mosquito dispersal between the islands and between the island and the mainland should be vigorously monitored.

## Background

Despite 50 years of malaria vector control efforts, malaria remains a major public health threat in tropical and subtropical countries [1-3]. In recent years, malaria has caused increased human mortality and morbidity as malaria epidemics have spread to areas where it was previously rare [4,5]. The current strategies for malaria control involve the treatment of infected individuals with antimalarial drugs to kill the parasites and vector management to reduce human-vector contacts via residual spraying and the use of insecticide-impregnated bednets. As demonstrated in multisite trials throughout Africa, the large-scale use of insecticide-treated bednets can reduce overall mortality by up to 30% [6] and morbidity in young children [7]. The emergence of insecticide resistance in mosquito vectors [8] and antimalarial drug resistance in *Plasmodium *[9] has significantly reduced the viability of many malaria control programs. An efficacious malaria vaccine will not be available in the near future [10].

One potential alternative malaria control strategy is based on the genetic disruption of mosquito vector competence [11-13]. This genetic control approach requires identification and cloning of parasite-inhibiting genes in the mosquito vectors, development of stable and efficient mosquito transformation tools and the development of strategies for spreading the parasite-inhibiting genes. Over the past several years, remarkable progress has been made in the development of mosquito germline transformation and in the identification of parasite-inhibiting molecules. For example, *A. gambiae *cell lines were successfully transformed with the *Hermes *element [14,15], and the *Minos *transposable element bearing an exogenous gene was efficiently integrated into the genome of *Anopheles stephensi *[16,17]. Genetic linkage maps have been constructed for *A. gambiae *[18], and genes conferring mosquito refractoriness to malaria parasites have been mapped [19]. Availability of complete *A. gambiae *genome sequences will greatly facilitate identification and cloning of parasite-inhibiting genes [20].

The success of the transgenic mosquito approach depends on the spread and even fixation of parasite-inhibiting genes into natural populations. Presently, releasing transgenic mosquitoes to the field is premature. Isolated islands have been suggested as an ideal natural site for testing transgenic mosquito release strategies and spatial spreading of transgenes [13,21,22]. Information on mosquito population genetic structure and gene flow on islands and the surrounding mainland area is critical. Using microsatellite markers, the *A. gambiae *population genetic structure in the African continent has been examined [23-28]. These studies revealed that the Great Rift Valley in East Africa is a substantial gene flow barrier for *A. gambiae*; however, no significant genetic structure was detected for mosquito populations between western Kenya and West Africa. The minimum area associated for a deme of *A. gambiae *in western or coastal Kenya is larger than 50 km [24]. Simard *et al*. [29] found a high degree of genetic differentiation of the *Anopheles arabiensis *populations from the high plateau of Madagascar and those from Réunion and Mauritius islands (F_ST _ranges from 0.080 to 0.215). Population substructure was also detected on the island of São Tomé, West Africa [22].

The present study examined the genetic diversity and the population genetic structures of *A. gambiae *mosquitoes from five islands on Lake Victoria and the surrounding mainland in western Kenya. This information is valuable for selecting field sites to test transgene release strategies and evaluating the spread of transgenes in nature.

## Materials and Methods

### Study sites and mosquito collection

**Figure 1:**
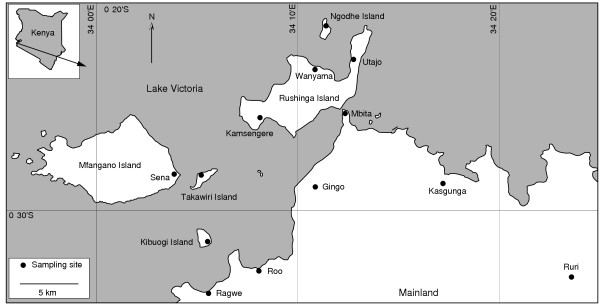
Map of study area showing the distribution of *Anopheles gambiae *populations on the Lake Victoria islands and the surrounding mainland area in Suba District, western Kenya.

Anopheline female mosquitoes were collected from seven villages on five islands in Lake Victoria and from six villages in the mainland Suba District, western Kenya (Fig. 1). The sampled islands were Kibuogi, Mfangano (Sena village), Ngodhe, Takawiri and Rushinga. Mosquitoes were collected from three villages (Kamsengere, Utajo and Wanyama) on Rushinga Island and one village on each of the other islands. Mfangano Island is the largest and the most offshore (about 10 km away from the nearest mainland village). Rushinga Island is the most populated among the five islands and is connected to the mainland by a walkway. The islands are about 2.5–21.0 km apart. Also, five mainland villages (Ragwe, Roo, Gingo, Mbita and Kasunga) along the shore of Lake Victoria and one inland village (Ruri) about 11 km away from the lakeshore were selected. The distance between the islands and the mainland sites ranges from 4.9 to 35.1 km. Malaria on these islands and the mainland area is holoendemic, and *A. gambiae *mosquitoes are the major malaria vectors in this region [30].

At least 170 anopheline mosquitoes were collected from four to 28 houses within each village using the pyrethrum spray collection method [31]. Mosquitoes from Mbita, Kasgunga and Ruri were sampled in May 1997; collection in other villages was conducted in April and May 1999. *A. gambiae sensu lato *(*s.l*.) specimens were separated from other anophelines according to the identification key provided by Gillies and Coetzee [32] and then preserved in 95% ethanol and kept at -20°C until further analyzed.

### PCR assay for species identification

PCR analysis was conducted for species identification using the rDNA-PCR method because individual species within the *A. gambiae *species complex cannot be identified by morphology alone [33]. About 100 *A. gambiae s.l*. females per village were tested. If the initial PCR testing failed to amplify for a sample, then the PCR analysis was repeated once or twice until successful amplification was achieved. If a sample could not be identified after three PCR amplifications, it was scored as unknown.

### Microsatellite loci and genotyping

Six microsatellite markers were used for specimen genotyping, including AGXH1D1 and AGXH131 of Chromosome X, AG2H46 and AG2H79 of Chromosome 2, and AG3H29C and AG3H33C of Chromosome 3 [17,18,22,23]. Microsatellite analyses were conducted on 51–70 individuals per village (See the additional file: A table of sample size, allelic number, heterozygosities and breeding coefficient of 13 *A. gambiae *populations from the Lake Victoria islands and the surrounding mainland in western Kenya).

A Li-Cor Model 4200 Automated DNA Analyzer (Li-Cor Inc., Lincoln, NE) was used for gel electrophoresis. For the apparatus to detect PCR products, one primer in every pair of microsatellite primers must be fluorescently labelled. To reduce the cost associated with synthesis of fluorescently labelled primers, we used the "tailed primer" method [34,35]; that is, the forward primer for each microsatellite locus was synthesized with an additional 19 bp sequence (5' CACGACGTTGTAAAACGAC 3') added to the 5' end of the primer. A third primer with the same 19 bp sequence was directly labelled with the fluorescence and was used as the sole type of labelled primer for the detection of all microsatellite alleles. The tailed primer method reduced the cost of oligonucleotide synthesis by >80%. The 10 μl PCR reaction contained 1X Taq buffer, 0.2 mM dNTPs, 1.5 pmol forward and reverse primers, 1.5 pmol fluorescently labelled 19 bp sequences, 1.5 mM MgCl_2_, 1.0 μg BSA, 1.0 unit *Taq *polymerase and about 20 ng genomic DNA. Cycling conditions in a MJ Research PTC-220 thermocycler were 35–40 cycles of 94°C for 30 seconds, 55°C for 30 seconds and 72°C for 45 seconds. Allele sizes were determined using Gene ImagIR computer software [36]. The allele sizes used in the analysis were true allele sizes that have been adjusted for the 19 bp tail in the forward primer.

### Data analysis

Microsatellite polymorphism was measured by the number of alleles and heterozygosity at each locus. Using the probability test available in the GENEPOP computer program [37], conformance with Hardy-Weinberg Equilibrium (HWE) was tested for each locus and population, and the Bonferroni correction was applied for multiple comparisons. The F_IS _statistics and probability test were used to determine whether distortion from HWE resulted from heterozygosity deficiency or excess using. Because the probability test is robust to low allele frequencies, rare alleles were not pooled. Variations in heterozygosity among the populations were analyzed following Weir's method [38], using the analysis of variance (ANOVA) with subpopulations, individuals, loci and interactions of loci, and individuals as factors. All factors were treated as random effects except loci. The Fisher exact test was performed to detect linkage disequilibrium for pair-wise loci in each population and the pooled population. Population genetic structure was examined with Wright's F-statistics (F_ST_) using FSTAT 2.8 [39]. F_ST _statistic appears to be more sensitive to detect intraspecific differentiation than R_ST _[40,41]. The standard deviations of the F-statistics were obtained for each locus by a jackknife procedure over all the alleles and were used to test the statistical significance. Nei's unbiased genetic distances [42] were calculated for all pairs of populations based on microsatellite allele frequencies at six loci using TFPGA [43]. A dendrogram was created based on the pair-wise genetic distances using the unweighted pair group method with arithmetic mean (UPGMA). The bootstrap confidence values were generated by 1,000 permutations.

The isolation-by-distance model of population genetic structure was tested by linear regression of pair-wise F_ST_/(1 - F_ST_) against the natural logarithm of straight-line geographical distance between population pairs [44]. Statistical significance of the regression was tested using the Mantel test with 10,000 permutations [45].

## Results

### Population genetic variability

A moderate to high level of polymorphism was found in six loci across the 13 populations (See the additional file). The three populations from Rushinga Island had a similar number of alleles per locus (ANOVA, F = 0.02, df = 2, P > 0.05) and observed heterozygosities (F = 0.029, df = 2, P > 0.05). Among the island populations, the average observed number of alleles per locus was not significantly different (F = 0.08, df = 6, P > 0.05), but observed heterozygosity varied significantly (F = 4.52, df = 6, P < 0.01). The three populations on Rushinga Island, Kamsengere, Utajo and Wanyama, showed significantly lower heterozygosity than other islands. Similarly, the six mainland populations did not differ in the number of alleles per locus (F = 0.29, df = 5, P > 0.05), but they varied significantly in the observed heterozygosities (F = 5.45, df = 5, P < 0.01). In particular, the Ruri population had the highest observed heterozygosity (0.343), about two-fold higher than the Mbita population (See the additional file). Overall, there was no significant difference between the island and mainland populations in the number of alleles per locus (7.3 vs. 6.8; t = 0.67, df = 74, P > 0.05) and observed heterozygosities (0.32 vs. 0.28; t = 1.82, df = 74, P > 0.05). A total of 12 private alleles were identified, nine of them from the island populations.

A total of 14.1% loci (11 out of 78 tests) showed significant departure from Hardy-Weinberg equilibrium, all due to heterozygote deficiency. This was caused entirely by heterozygote deficiency in the locus AG2H46, a locus known for the presence of null alleles in western Kenyan *A. gambiae *populations [46]. The Fisher exact test revealed linkage disequilibrium in 13 out of 195 pairs of loci (6.7%; data not shown), suggesting a low level of linkage disequilibrium among the six loci scored.

### Population genetic structure

A low, but significant, genetic structure was detected among the seven island and the six mainland populations (Table 1). The genetic differentiation in the seven island populations (F_ST _= 0.019, P < 0.001) was almost twice as high as the six mainland populations (F_ST _= 0.010, P < 0.001). Genetic differentiation between island and mainland populations was also small (F_ST _= 0.010, P < 0.001). Pair-wise comparisons between all populations revealed that only seven pairs (Kibougo/Kamsengere, Kasgunga/Kamsengere, Takawiri/Ruri, Sena/Ruri, Utajo/Ruri, Ngodhe/Ruri and Ngodhe/Gingo) exhibited significant F_ST _values, and six of them were between an island and a mainland population.

**Table 1:** F_ST _estimates of *Anopheles gambiae *populations on the islands of Lake Victoria and from surrounding mainland sites in western Kenya

Locus	Among seven island populations	Among six mainland populations	Between island and mainland areas	Among all populations
AGXH1D1	0.082***	0.022***	0.002***	0.042***
AGXH131	0.000	0.009***	0.005***	0.006***
AG2H46	0.008***	0.006	0.006***	0.009***
AG2H79	0.011***	0.011***	0.000	0.010***
AG3H29C	0.026***	0.008***	0.000	0.007***
AG3H33C	0.003***	0.009***	0.000	0.005***
Overall	0.019***	0.010***	0.003***	0.012***

* P < 0.05, ** P < 0.01, *** P < 0.001.

**Figure 2:**
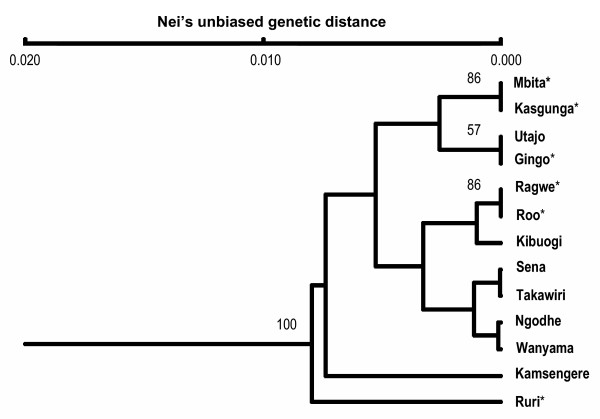
A UPGMA tree based on Nei's unbiased distance showing genetic divergence among *Anopheles gambiae *populations. The numbers above branches indicate those with >50% bootstrap support. The populations marked with an asterisk are the mainland populations.

The Mantel test revealed a significant correlation between geographic distance and pair-wise F_ST_/(1 - F_ST_) (P < 0.001), suggesting that the population genetic structure of *A. gambiae *populations from the island and mainland is consistent with the isolation-by-distance model. When the Ruri population is removed from the analysis, the correlation was still statistically significant (P = 0.015). Therefore, the population genetic structure of our study populations is consistent with the isolation-by-distance model. The cluster analysis revealed that the Ruri population, located farther inland from the other populations, was out-grouped from other populations with a significant bootstrap value, while other mainland and island populations were intermixed with non-significant branch bootstrap values (Fig. 2).

## Discussion

The present study demonstrated a similar level of genetic diversity between the island *A. gambiae *populations in the Lake Victoria and adjacent mainland populations in the Suba District, western Kenya. For the seven island populations, the average number of alleles at six microsatellite loci was 7.3 and the observed heterozygosity was 0.32. For the six mainland populations, the average number of alleles was 6.8 and the observed heterozygosity was 0.28. The population genetic diversity at most loci in this study was similar to other western Kenyan populations [23,47-49]. Compared with West Africa populations [23,27,48,49], lower heterozygosities, particularly at loci AG2H46, AG2H79 and AG3H33, were reported in this study, caused by fewer alleles detected in the studied populations. The comparable level of genetic diversity between island and mainland populations suggests that the island mosquito populations have a similar effective population size as the mainland populations, and they have not suffered severe genetic bottleneck during the previous vector control efforts. For each population, all loci except the AG2H46 locus did not show a significant deviation from Hardy-Weinberg equilibrium, suggesting that the microsatellite markers used in the study are not under strong selection and mosquito populations are in random mating. A heterozygote deficit at the locus AG2H46 was observed for all populations in this study. Heterozygote deficiency at the locus AG2H46 was also demonstrated in other western Kenya populations by Lehmann *et al*. [23,24]; the presence of null alleles as a result of mutations in the primer-annealing region was the cause.

A small but statistically significant genetic structure was detected for *A. gambiae *populations among the five islands in Lake Victoria (F_ST _= 0.019) and among the six villages in the mainland in an area of approximately 40 × 20 km^2 ^(F_ST _= 0.010). The degree of genetic differentiation between the island populations in this study was less than for the island *A. gambiae *populations of São Tomé, western Africa (F_ST _= 0.032) [22]. The lower F_ST _estimates in the populations in this study were probably caused by shorter distance between islands (3–15 vs. 23–38 km) [22] and a lack of mountainous topography as gene flow barriers. The F_ST _estimates for the mainland populations in this study were comparable to other studies on the western Kenya populations (F_ST _= 0.0033) [24,27]. The genetic differentiation between island and mainland populations was small but statistically significant (F_ST _= 0.003). Thus, there is a very small degree of genetic isolation between island and mainland populations. This estimation is consistent with the private allele distribution in the studied populations, in which nine of the 12 private alleles were from the island populations. Further evidence for a small degree of genetic differentiation between island and mainland populations is from pair-wise population comparisons in which six out of the seven pairs that exhibited significant genetic differentiation were between an island population and a mainland population.

The low level of genetic differentiation between island and mainland mosquito populations implies large gene flow between the two areas (83.1 migrants per generation). The normal flight range of *A. gambiae *is usually less than 1 km [50]. The distance to the lake shore of the mainland from the islands ranges from 2.5 to 15 km, farther than the normal flight range of the mosquitoes. Thus, mosquito migration is likely assisted by wind. Lindsay *et al*. [51] found that the spatial distribution of *A. gambiae *mosquitoes was related to the predominant wind direction at night, suggesting that wind assisted the dispersal of mosquitoes from their breeding site. *A. gambiae *have been shown to fly up to 7 km with the assistance of wind [52,53]. This distance is in the range for mosquitoes to disperse between the closest islands and between islands and their closest mainland in this study area. Mosquitoes may also use one island as a stepping-stone to extend their dispersal distance.

Mosquito migration may also be assisted by human activities. A study on *Aedes polynesiensis *populations from islands found no significant effect of geographic distance on the population genetic structure, but detected a significant correlation between gene flow and commercial traffic by planes and/or boats between islands [54]. The introduction of *A. arabiensis *to the Mascarene islands and Madagascar was thought to be caused by human transportation by steamship lines [55,56]. In Lake Victoria, small wooden boats may transport mosquito larvae between the islands and the mainland. *A. gambiae *larvae were collected at the bottom of a wooden fishing boat [57]. Rushinga Island in the study area was connected to the mainland by a walkway, and the island mosquito larvae could be moved to the mainland by vehicle transportation.

The results of this study of the population genetic stricture of island and mainland *A. gambiae *populations have implications for the ecological safety evaluation of the transgenic mosquito release program. During the initial field test of environmental safety and public health consequences by transgenic mosquito release, ideal sites would be islands that are totally genetically isolated from other islands and the mainland, with a sufficient number of human inhabitants and active malaria transmission on the island. Such an island may be extremely difficult to find, so islands with some genetic isolation from the mainland may have to be chosen. If so, the Lake Victoria islands could be used as field test sites; however, due to potential gene flow between the islands and between the islands and the mainland, mosquito dispersal between the islands and between the islands and the mainland should be vigorously monitored. After the release of the genetically modified mosquitoes, long-term monitoring programs should be launched to evaluate the spread of the transgenes to any unintended areas. In addition, methods to minimize the negative effects of transgene leak need to be developed prior to the field trial of transgene release [58].

## Conclusions

This study showed that a low level of genetic differentiation existed between the island and mainland populations and no any genetically-isolated population was found among the 13 mosquito populations. If the islands on Lake Victoria were used as a trial site for the program to release genetically-modified mosquitoes, short-term and long-term mosquito dispersal between the islands and between the island and the mainland should be vigorously monitored.

## Authors' Contributions

HC conducted species identification using PCR, microsatellite analyses and drafting the manuscript. NM was responsible for sample collection, and participated in species identification and drafting the manuscript. JB and GY supervised the study, and assisted data analysis and manuscript preparations.

## Supplementary Material

Additional File 1 A table of sample size, allelic number, heterozygosities and breeding coefficient of 13 *A. gambiae *populations from the Lake Victoria islands and the surrounding mainland in western Kenya. Click here for file
