# Supplementary material for: Population genetic structure of Anopheles gambiae mosquitoes on Lake Victoria islands, west Kenya
Source: Malar J. 2004 Dec 6;3:48. doi: 10.1186/1475-2875-3-48 (PMC543573; doi:10.1186/1475-2875-3-48)
Supplement: Additional File 1 — A table of sample size, allelic number, heterozygosities and breeding coefficient of 13 A. gambiae populations from the Lake Victoria islands and the surrounding mainland in western Kenya. [file 1475-2875-3-48-S1.PDF]

Table Sample size (in parentheses), allelic number (n), observed (H_o) and expected (H_e) heterozygosities and breeding coefficient (F_{IS}) of 13 *Anopheles gambiae* populations from the Lake Victoria islands and the surrounding mainland in western Kenya

Chromosome	Locus	Kibuogi (61)				Takawiri (69)				Sena (65)				Kamsengere (55)			
		n	H_o	H_e	F_{IS}	n	H_o	H_e	F_{IS}	n	H_o	H_e	F_{IS}	n	H_o	H_e	F_{IS}
X	AGXH1D1	3	0.270	0.266	-0.014	5	0.300	0.278	-0.082	6	0.330	0.313	-0.055	5	0.190	0.206	0.078
	AGXH 131	10	0.410	0.412	0.004*	11	0.460	0.457	-0.006	10	0.430	0.446	0.037*	10	0.320	0.363	0.119
2	AG2H 46	13	0.410	0.497	0.177**	11	0.450	0.576	0.220***	9	0.280	0.500	0.442***	9	0.240	0.384	0.378***
	AG2H 79	4	0.320	0.334	0.043	4	0.340	0.430	0.210	5	0.370	0.372	0.006	4	0.240	0.235	-0.021
3	AG3H 29	2	0.310	0.260	-0.197	2	0.370	0.283	-0.311	4	0.380	0.320	-0.188	3	0.290	0.218	-0.333
	AG3H 33	13	0.380	0.393	0.034	13	0.370	0.450	0.179	13	0.390	0.457	0.149*	10	0.360	0.322	-0.121
	Average	7.5	0.350	0.360	0.028	8	0.382	0.412	0.069*	7.8	0.363	0.401	0.097*	6.8	0.273	0.288	0.050

Chromosome	Locus	Wanyama (51)				Utajo (52)				Ngodhe (70)				Ragwe (60)			
		n	H_o	H_e	F_{IS}	n	H_o	H_e	F_{IS}	n	H_o	H_e	F_{IS}	n	H_o	H_e	F_{IS}
X	AGXH1D1	5	0.180	0.212	0.152	5	0.230	0.232	0.009	6	0.340	0.341	0.003	3	0.300	0.268	-0.121
	AGXH 131	8	0.290	0.332	0.127	10	0.270	0.305	0.115	11	0.490	0.550	0.110	9	0.390	0.420	0.073
2	AG2H 46	9	0.210	0.282	0.259*	10	0.280	0.339	0.176*	10	0.350	0.558	0.375***	11	0.310	0.465	0.335***
	AG2H 79	5	0.220	0.268	0.182*	4	0.240	0.271	0.116	4	0.390	0.413	0.057	5	0.300	0.333	0.100
3	AG3H 29	3	0.170	0.161	-0.059	2	0.240	0.190	-0.268	2	0.210	0.216	0.026	3	0.280	0.234	-0.197
	AG3H 33	10	0.320	0.297	-0.079	11	0.340	0.331	-0.028	13	0.440	0.511	0.140	13	0.400	0.419	0.045
	Average	6.7	0.232	0.259	0.111*	7	0.267	0.278	0.041	7.7	0.370	0.432	0.142*	7.3	0.330	0.357	0.074*

Chromosome	Locus	Roo (56)				Gingo (54)				Mbita (54)				Kasgunga (56)			
		n	H_o	H_e	F_{IS}	n	H_o	H_e	F_{IS}	n	H_o	H_e	F_{IS}	n	H_o	H_e	F_{IS}
X	AGXH1D1	3	0.240	0.236	-0.016	5	0.230	0.245	0.063	3	0.150	0.135	-0.113	3	0.220	0.238	0.075
	AGXH 131	9	0.360	0.361	0.003	9	0.350	0.316	-0.108	7	0.220	0.220	0.002	10	0.380	0.396	0.042
2	AG2H 46	11	0.230	0.380	0.398***	11	0.210	0.330	0.367***	7	0.150	0.231	0.354***	9	0.280	0.374	0.253***
	AG2H 79	4	0.320	0.302	-0.061	5	0.190	0.225	0.157*	4	0.180	0.202	0.112	4	0.290	0.315	0.081
3	AG3H 29	4	0.310	0.214	-0.454	3	0.160	0.183	0.125	2	0.110	0.147	0.256	2	0.200	0.233	0.142
	AG3H 33	8	0.280	0.294	0.048	9	0.360	0.344	-0.049	8	0.200	0.223	0.104	14	0.380	0.426	0.110*
	Average	6.5	0.290	0.298	0.031	7	0.250	0.274	0.096*	5.2	0.168	0.193	0.135*	7	0.292	0.330	0.123*

Chromosome	Locus	Ruri (64)			
		n	H_o	H_e	F_{IS}
X	AGXH1D1	3	0.290	0.288	-0.008
	AGXH 131	10	0.430	0.485	0.113*
2	AG2H 46	12	0.320	0.409	0.219***
	AG2H 79	4	0.310	0.290	-0.069
3	AG3H 29	3	0.250	0.279	0.104
	AG3H 33	13	0.460	0.401	-0.149
	Average	7.5	0.343	0.359	0.039

*P < 0.05; **P < 0.01; ***P < 0.001.
